# Integrating nonpharmacologic strategies for pain with Inclusion, Respect, and Equity (INSPIRE): a digital health study protocol for a pragmatic multisite randomized controlled trial

**DOI:** 10.1186/s13063-025-09402-8

**Published:** 2026-01-08

**Authors:** Jesse Ristau, Agatha Okobi, Matthew J. Miller, Jing Cheng, Sibel Deviren, Eleanor Bimla Schwarz, Tess Fairchild, Janice Y. Tsoh, Adrian Aguilera, Cindy J. Zheng-Huang, Jason M. Satterfield

**Affiliations:** 1 Department of Medicine, Division of General Internal Medicine, University of California, 1701 Divisadero St., Suite 500, San Francisco, CA 94115 USA; 2 Department of Physical Therapy and Rehabilitation Science, University of California, San Francisco, CA USA; 3 School of Social Welfare, University of California, Berkeley, CA USA; 4 Department of Psychiatry and Behavioral Sciences, University of California, San Francisco, CA USA; 5 Department of Preventive and Restorative Dental Sciences, University of California, San Francisco, CA USA; 6 Department of Orthopedic Surgery, Spine Center, University of California, San Francisco, CA USA

**Keywords:** Chronic pain, Digital health, Health coaching, Health equity, Cognitive behavioral therapy, Mindfulness, Movement

## Abstract

**Background:**

Chronic pain (CP) is among the most common medical conditions, is the leading cause of disability, and is often refractory to medical treatment. The CDC and the American College of Physicians recommend nonopioid and nonpharmacologic treatment as first-line strategies, including cognitive-behavioral therapy (CBT), mindfulness-based interventions (MBI), and movement-focused interventions (MFI); however, only 3% of people with chronic pain (CP) have access to these evidence-based treatments. Patients from historically minoritized populations and those speaking languages other than English are particularly underserved and may go untreated. Stigma regarding CP and interventions to manage it present additional obstacles.

**Methods:**

The Integrating Nonpharmacologic Strategies for Pain with Inclusion, Respect, and Equity (INSPIRE) study evaluates a blend of these non-pharmacologic, evidence-based interventions using a pragmatic, parallel, two-arm RCT comparing intervention participants with wait-list controls. The INSPIRE intervention includes a 12-week curriculum delivered via a multilingual mobile app paired with weekly telehealth coaching. The primary outcome will be the “Pain, Enjoyment, and General Activity (PEG)” scores at 3 months. Secondary outcomes include PEG scores at 6 and 12 months, physical functioning, quality of life, sleep, depression, anxiety, and global satisfaction with treatment at 3, 6, and 12 months. Primary mediators to be examined include pain cognitions and health behaviors. Secondary mediators may include stigma and isolation, trust in healthcare providers, and both engagement and satisfaction with the INSPIRE intervention. The intention-to-treat primary analysis will be conducted with covariates including baseline measures of pain, anxiety, and physical function.

**Discussion:**

The full RCT protocol and intervention details are provided following the SPIRIT guidelines. Implications for improving treatment access via culturally and linguistically tailored digital health interventions are discussed along with the potential for health coaching to drive mobile app engagement.

**Trial registration:**

ClinicalTrials.gov ID NCT06183281. Trial registration data set can be found here: https://clinicaltrials.gov/study/NCT06183281 and in Additional file 1.

**Supplementary Information:**

The online version contains supplementary material available at 10.1186/s13063-025-09402-8.

## Background

### Unmet clinical need

Only 3% of people with chronic pain (CP) have access to evidence-based, non-pharmacologic, integrative treatments even though CP is among the most common and substantial diseases with an estimated incidence higher than that of diabetes, cancer, and heart disease combined [1–11]. CP substantially impacts physical and mental functioning, productivity, and quality of life; it is the leading cause of disability and is often refractory to medical treatment [3, 12, 13]. The CDC and the American College of Physicians recommend nonopioid and nonpharmacologic treatment for CP given the strong evidence for mind-body interventions such as cognitive-behavioral therapy (CBT), mindfulness-based interventions, and physical therapy (PT) and the recognition of harms caused by the inappropriate use of opioids for CP [12, 14–21].

### Health and healthcare disparities for pain

Health disparities populations (HDPs) (e.g., Black/African American (B/AA), Latinx) are more likely to have their pain underestimated by providers, are less likely to receive comprehensive diagnostic workups, are less likely to receive opioids, and receive less aggressive pain treatment [22, 23]. Patient-provider language concordance influences reported pain intensity and management plans for Latinx and Chinese patients [24]. Under-reporting of pain has been tied to cultural beliefs such as stoicism or desiring to be a good patient [25, 26]. Other patient-level factors include distrust in clinicians, internalized stigma, social isolation, and low expectations of treatment. Provider-level factors include implicit biases that manifest in poor decision making, failed treatment coordination, and poor communication. Health-system factors include less access to services and less availability of treatments like CBT or PT, particularly for HDPs [27–29].

Stigma is a fundamental cause of health disparities [30, 31]. Stigma contributes to disparities by creating obstacles to socioeconomic resources (e.g., education) and by initiating a cascade of psychological, behavioral, and biological responses that undermine health and contribute to social isolation [30, 32]. Low-income minorities who experience stigma based on race, poverty, or medical diagnoses (e.g., psych, substance use disorders (SUD)) will have greater difficulty accessing and accepting CP treatments unless the stigma(s) is also addressed.

#### The need for scalable innovations to improve access

CBT is considered the gold standard for CP management across diverse populations, although most patients in need do not receive it [18, 27, 28, 33–35]. Acceptance and mindfulness-based interventions (MBI) have also received broad support, but access has been even more limited [27, 28, 36–43]. Newer “third wave” psychotherapies such as Acceptance and Commitment Therapy and mindfulness-based cognitive therapy have blended traditional CBT with MBI, creating important new tools in need of innovative plans to improve access [27].

Movement-focused interventions (MFI) include stretching, strengthening, and mobility exercises to improve function, physical conditioning, disability, and health. Despite MFI being considered part of gold-standard treatment for most CP conditions, patient motivation and adherence are highly problematic [44–48]. MFI has also been successful blended with CBT, MBI, and third wave psychotherapies, but further investigation is needed [49, 50]. Additionally, a recent comprehensive review of the chronic pain management literature identified the pressing need for more pragmatic RCT’s, innovative implementation strategies, cultural/linguistic adaptations, and the exploration of digital tools that extend reach and reduce cost [27, 28].

#### Imagining a HDP-inclusive digital solution

Digital health has received support as a means of assessing and monitoring patients, optimizing integrative interventions, and improving CP outcomes [51–55]. Both internet and app-delivered interventions have demonstrated efficacy for depression, anxiety, and pain but have rarely been culturally or linguistically adapted [56–62]. Digital pain assessments may lower stigma and improve accuracy while digital CBT and MBI substantially increase reach, reduce cost, and remove other barriers to treatment [56, 57, 63–65]. Virtual MFI has strong potential to increase rehabilitation access and has been shown to be as effective as in-person MFI [66–69]. As with most digital interventions, sustaining patient engagement can be challenging. Preliminary studies have shown that hybrid models that blend coaching with digital CBT/MBI/MFI can improve engagement while maintaining positive outcomes even when the clinician contact is modest [70–76].

Based on prior experiences, both providers and patients may assume that digital health interventions will be unable to address the social isolation and need for social support often found in CP patients with complex social histories. Fortunately, the inclusion of a personal telehealth coach and structural features such as digital discussion forums, live (online) meditation groups, zoom support groups, personal text messages (even when automated), and linkages with the primary care team can still produce a sense of connection and rapport while maintaining the efficiency, lower cost, and greatly improved access to evidence-based care [77, 78].

Most current CP apps have not been validated, are not integrative, and have not taken cultural, linguistic, or digital literacy needs into account. Moreover, sustaining engagement and reducing social isolation with a mobile app has proven difficult without the concurrent support of a health care provider or social network. More efficient and scalable solutions with linguistic and cultural adaptations are needed to serve a diverse population with CP. The INSPIRE intervention has the potential to be a cost-effective alternative to the current standard of care which leaves many patients with suboptimal pain control.

#### Objective

We developed a culturally tailored, trilingual mobile phone app that blends CBT, MBI, and MFI delivered over 12 weekly modules and supported by weekly health coaching to address non-malignant chronic pain in adult primary care patients. This Phase III clinical trial will assess the effectiveness of this intervention and examine potential mediators. We hypothesize that INSPIRE participants will show significantly greater reductions in PEG scores (i.e., pain intensity, interference of enjoyment, and reductions in general activities) from baseline to 3 months (i.e., primary outcome) in comparison to waitlist controls with additional assessments at 6 and 12 months. Secondary outcomes will include the HEAL CDE measures and analysis of potential primary mediators (e.g., catastrophizing, self-efficacy, psychological flexibility) and secondary mediators (e.g., trust, lower stigma).

## Methods

### Trial design

The efficacy of the INSPIRE intervention will be determined by a multisite, parallel, two-arm pragmatic RCT comparing intervention participants with waitlist controls over a study period of 12 months. The primary outcome will be PEG scores at 3 months with secondary outcomes including PEG at 6 and 12 months, and physical functioning/quality of life, sleep, depression, anxiety, and global satisfaction with treatment at 3, 6, and 12 months. Secondary analyses will also determine if primary mediators of pain cognitions (catastrophizing, psychological flexibility, pain self-efficacy) and health behavior (behavioral activation, exercise, movement) and secondary mediators of stigma and isolation, trust in healthcare, and satisfaction with the INSPIRE intervention play a role in improved pain outcomes.

**Fig. 1:**
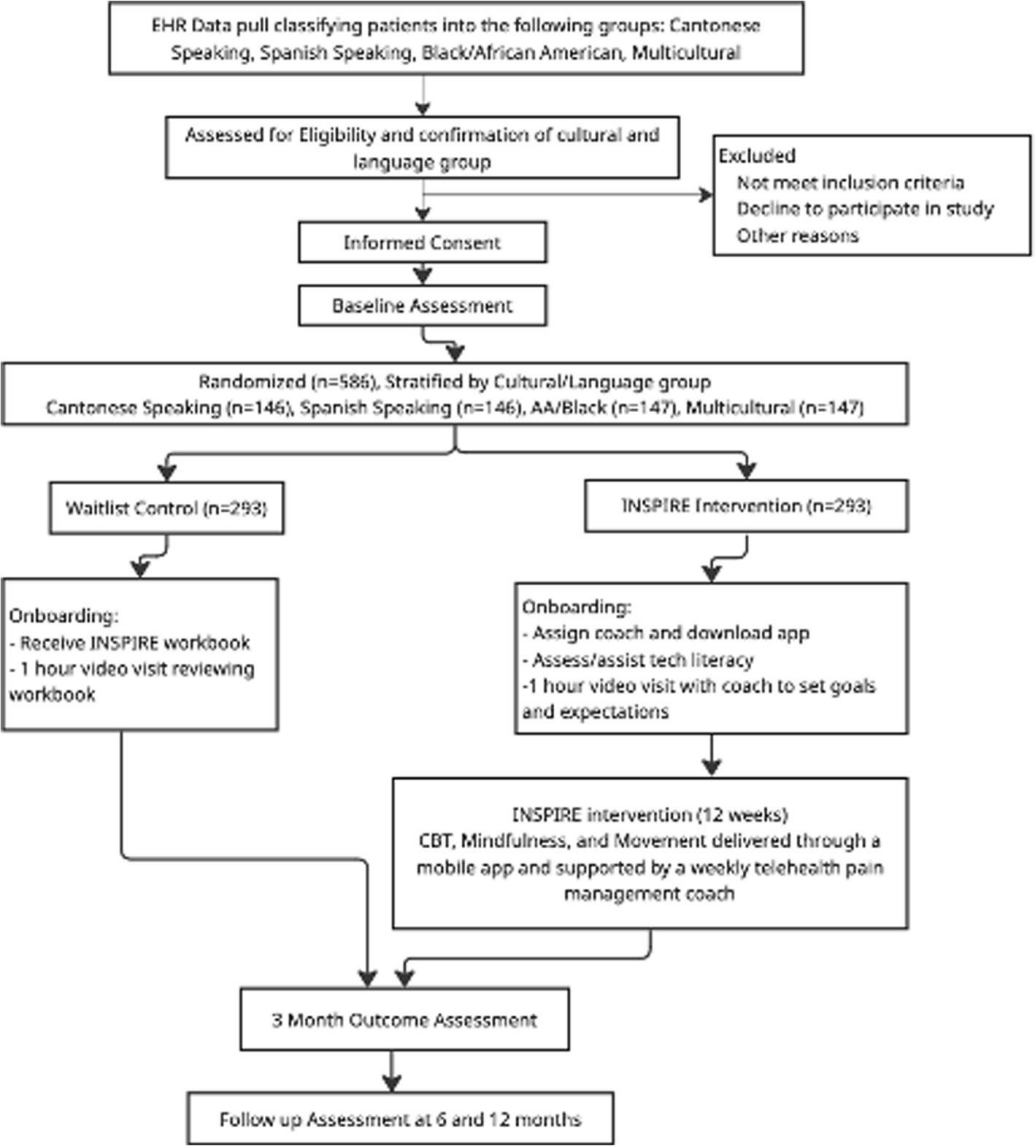
Flowchart of the INSPIRE study

The feasibility and acceptance (F&A) of this research protocol was first assessed in a 3-month, single-arm pilot of *n*=24 participants (8 from each language group). F&A outcomes included the success of participant recruitment and retention, patient adherence, intervention fidelity, data collection tools and schedule, and participant satisfaction. Feedback on the trial methodology and the mobile app was collected and used for optimization prior to the start of the RCT. The study protocol is summarized in the flowchart (Fig. 1). The Study protocol adheres to the Standard Protocol Items: Recommendations for Interventional Trials (SPIRIT) 2025 guidelines [79]. The SPIRIT Checklist can be found in Additional file 2, and the “SPIRIT Figure” study timeline can be found in Table 1.

**Table 1:** “SPIRIT figure” study timeline

	Trial Period						
	Enrollment		Post-randomization				
**Timepoint**	Screening	Baseline	3mo	5mo	6mo	11mo	12mo
**ENROLLMENT**							
Eligibility Screen	X						
Informed consent	X						
Randomization		X					
**INTERVENTION or comparator**							
INSPIRE Intervention		X	X	X		X	
Waitlist Control		X					
**ASSESSMENTS**							
Primary Outcome							
PEG [80]		X	X		X		X
Secondary outcomes							
Quality of life (WHOQOL)		X					X
PROMIS Physical functioning 6b [81]		X	X		X		X
PROMIS Sleep Disturbance 6a [82]		X	X		X		X
Sleep duration [83]		X	X		X		X
PHQ-8 (Depression) [84]		X	X		X		X
GAD-7 (Anxiety) [85]		X	X		X		X
Global Impression of Change [86]			X				X
TAPS-1 & TAPS-2 (Substance Use) [87]		X	X		X		X
Primary Mediators							
Pain Catastrophizing [88]		X	X		X		X
Psychological Inflexibility [89]		X	X		X		X
Pain Self-Efficacy [90]		X	X		X		X
Behavioral/Social activation [91]		X	X		X		X
Secondary Mediators							
PROMIS Social isolation 8a [92]		X					X
Internalized Stigma [93]		X					X
Healthcare Relationship Trust [94]		X					X
Adherence/Engagement			X				X
Mobile App Rating Scale [95]			X				
Other Covariates							
Demographics		X					
Digital Health Literacy [96]		X					

### 2.2 Study setting

This multisite study recruits from adult primary care clinics that represent two distinct health care systems located in a diverse, urban setting, the San Francisco Bay Area. These systems include a large academic health center (UCSF Health) and a network of community safety net clinics for the urban underserved (San Francisco Health Network; SFHN) managed by the San Francisco Department of Public Health (SFDPH). At UCSF Health, patients are majority non-White: 47% White, 24% Asian, 10% Latino, 9% African-American, and 10% other ethnicities. The payor mix is diverse, with 53% private insurance, 30% Medicare, 13% Medi-Cal, and 4% self-pay. SFHN serves 125,000 diverse patients and is comprised of 10 community safety net hospitals, including clinics which serve majority Chinese, Latinx, and Black/African American patients. At SFHN, patients served in the outpatient setting are majority non-White: 13% White, 23% Asian, 43% Hispanic/Latinx, and 13% Black/African American, and a majority have public insurance: 60% Medi-Cal, 23% Medicare, 3% private, and 5% uninsured. The INSPIRE intervention is delivered via mobile app or web browser using existing patient devices. The health coaching element is entirely remote, taking place over scheduled Zoom video visits. In the event of wifi or other technical difficulties, coaching will be delivered by telephone.

### Participants

#### Participant eligibility

Participants must be (1) age 18 or older; (2) speak English, Spanish, or Cantonese; (3) have a UCSF Health or SFHN PCP; (4) be willing to use a smartphone (iOS or Android – either their own or one provided by the study); (5) have chronic, non-malignant pain for at least 3 months; and (6) be willing to participate in a 12-month patient-centered CP management study where they will be randomized into one of two comparison arms. Spanish and Cantonese languages were chosen in addition to English due to their high prevalence in the San Francisco Bay Area. Any individuals with conditions that prevent informed consent (e.g., dementia, active psychosis) and patients with chronic pain-related surgery scheduled in the next 12 months will be excluded. Patients will not be excluded if they have a current SUD, mood disorder, or anxiety disorder. Participants are allowed to participate in other concomitant pain management interventions, including procedural interventions (e.g., joint injections), during the 12-month study but are asked to report these treatments. Pharmacotherapy interventions (e.g., opioids) are not assessed due to patient concerns voiced during the intervention development phase regarding potential unwanted changes to their medication regimen that could result from study participation.

##### Recruitment: Participant identification, screening, and consent

Study clinical research coordinators (CRCs) will utilize a HIPAA partial waiver of consent to identify potentially eligible patients using ICD-10-CM codes in the electronic health record [97, 98]. ICD-10 codes include those commonly used for chronic pain as well as more specific codes that often indicate conditions that cause chronic pain (e.g., low back pain, osteoarthritis, spinal stenosis, etc.). PCPs will be sent lists of their identified patients and asked to indicate those that should not be contacted for study recruitment. The remaining potentially eligible patients will be screened for eligibility by telephone by culturally and linguistically concordant staff. Those who consent to participate will complete baseline measures and be randomized. Participants may receive up to $270 for participation over the 12-month study period based on completion of each timepoint assessment.

#### Participant randomization

We will randomize at the level of the patient. While this poses a modest risk of provider contamination between intervention and control participants, providers will not receive any communication from the study team after enrollment. We have further addressed the potential effect of contamination by increasing sample size, allowing detection of smaller intervention effects.

Participants will be stratified by 4 demographic groups (Cantonese-speaking, Spanish-speaking, Black/African American English-speaking, and multi-cultural English-speaking) and randomly assigned 1:1 to either the INSPIRE intervention (Group 1) or control groups (Group 2). The CRC will utilize random block randomization to maximize balance between treatment groups throughout accrual while ensuring the randomization sequence remains unpredictable. A random allocation sequence was generated using a computer, where treatment groups were labeled as Group 1 or 2. After participants consent to study participation and complete baseline measures, clinical research coordinators will assign the next available study ID number in sequence, identify the allocated group, and inform participants of their assignment. The study ID number and Personal Health Information (PHI) will be stored in a HIPAA-compliant manner (REDCap) along with the intervention assignment (Group 1 or 2). Study personnel responsible for outcome evaluations will be blinded to which Group 1 or 2 represents.

### Blinding

Study personnel responsible for survey administration and/or data analysis will be blind to participant condition and are not unblinded. Participants will not be blinded as it was not possible to create a “sham” digital intervention and coaching program.

### Study onboarding

When participants assigned to Group 1 (INSPIRE intervention) are notified of their assignment, they receive language concordant email instructions regarding how to download and access the INSPIRE mobile application from the Apple or Google Play (i.e., Android) app stores and are instructed how to access the INSPIRE intervention website using any standard browser for participants who prefer a website to an app. For those in Group 1, a CRC also emails a brief biography of their assigned health coach. The assigned coach calls the participant to schedule the Week 0 session which is held on a remote video platform (Zoom) and reminds them to download and login to the app before the Week 0 meeting. To better support and engage patients with limited tech literacy, the Digital Health Literacy Instrument will be administered at baseline to identify Group 1 participants that may need extra technical support [96]. For those with low digital literacy or if the participant demonstrates technical issues with the app or is unfamiliar with Zoom during the onboarding session, the coach provides brief technical support and troubleshooting over the phone. If additional support is needed, the coach schedules a tech support meeting with the participant and the CRC who uses instructional videos and 1×1 “tech tutoring” sessions.

For participants assigned to Group 2 (waitlist controls), the CRC emails the INSPIRE Workbook pdf or mails a print copy if requested. Onboarding for Group 2 will include sharing and introducing the INSPIRE intervention manual (print or pdf) via a one-time hour-long video session. CRCs call the participant to schedule the workbook orientation, walk through the table of contents, and encourage participants to explore and use the manual on their own.

### Trial interventions

#### INSPIRE Intervention: conceptual background/model

**Fig. 2:**
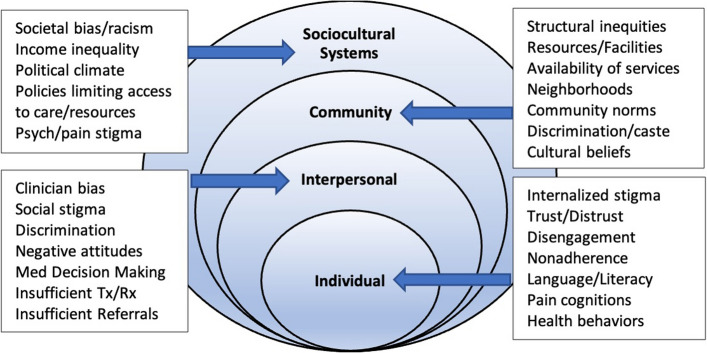
Social-ecological model of chronic pain

INSPIRE’s conceptualization of CP is grounded in the biopsychosocial (BPS) model that highlights the interplay of biological (e.g. genetic vulnerability, physical health, tissue damage), psychological (e.g. emotional distress, attention, cognition), and social factors (e.g. social location, social determinants of health, culture) [27]. Stigma, bias, and other drivers of disparities are part of the BPS model which informed the development of this intervention. The social-ecological model of CP shown in Fig. 2 highlights select variables thought to play a role in disparities and influenced by the INSPIRE intervention.

#### Intervention development

Throughout the development process, we applied a human-centered design (HCD) approach that focuses on the usability and needs of those the intervention is meant to serve including B/AA, Latinx, and Chinese patients. Digital health solutions that use HCD to incorporate the patient and clinician voice and account for their workflows can augment digital tools to deliver high-quality and personalized care in routine practice and can be particularly valuable in designing interventions for HDPs [99–108]. We first used stakeholder focus groups applying HCD principles to create the INSPIRE patient-facing app and data collection tool. Brief alpha and beta testing occurred after the formative focus groups, followed by a 3-month feasibility and acceptability pilot to further test the app functionality and tailoring. As a final assessment of intervention fidelity to our evidence-based interventions (i.e., CBT, MBI, MFI), we assembled an independent “Clinical Science and Practice Advisory Board” to review and approve the content. This board was comprised of local pain management clinicians, including a pain medicine physician, psychologist, pharmacist, physical therapist, and the director of the ZSFG pain management service.

#### INSPIRE intervention: mobile app

The INSPIRE intervention includes a participant-facing mobile app that delivers 12 weekly, integrative treatment modules which combine aspects of CBT, MBI, MFI, and a personal, telehealth coach who will meet with the participant weekly for 3 months. App content also includes an education “library,” tools for “quick relief” (i.e., acute exacerbations of pain), a community bulletin board, and a “favorites” section that the participant and coach will use to create a tailored pain management plan. All INSPIRE intervention participants will receive “booster sessions” with their coach at months 5 and 11 to review pain management skills and re-visit key modules within the mobile app. Throughout the 12-month study, intervention participants can complete and view their own patient-reported outcome (PRO) data (e.g., PEG, PHQ-8, GAD-7) to self-monitor their progress via the INSPIRE app. A brief outline of the 12 modules can be found in Table 2. A screenshot of the app’s homepage is listed in Additional file 3.

**Table 2:** INSPIRE intervention modules

**Session**	**Topic**	**Focus of coaching session**
0	Introduction	Introductions, building rapport, identifying goals
1	Mind-body and pain introduction	Introduction of nonpharmacologic interventions, rapport building, norm setting
2	Mindfulness and CBT	Mindfulness skills, CBT introduction (“Thinking about Thinking”)
3	Movement	Movement education, mind and movement, exercise
4	Stress and Chronic Pain	Definition of stress, coping skills, getting out of your head
5	CBT Deep Dive #1	“ABC” Model; Thoughts and feelings, behaviors and feelings
6	CBT Deep Dive #2	Cognitive restructuring and mastering thoughts, flexibility; ABCD exercises
7	Sleep	Sleep education, insomnia and chronic pain, sleep assessment; sleep meditations
8	Building Self-Efficacy for Pain Management	Self-efficacy, self-compassion; Loving-Kindness Meditation
9	Mindfulness and Movement	Catastrophizing and mindfulness, exercise and aerobic training, movement meditations
10	Social Connections and Community	Social support, isolation and loneliness, support circle, mindful communication
11	Building your Toolbox	Gratitude, Yoga, Tai Chi, positive emotions, savoring
12	Reinforcing skills and practicing for the future	Building/revising pain plan, review key skills, self-care/compassion, guided reflection, find your community

Outlines the focus and topic of each coaching session delivered to participants during the 12-week intervention period, including the baseline session at Week 0. Each weekly session covered a distinct theme aligned with the study’s intervention goals. The content was structured to support behavior change and skill development relevant to the primary and secondary outcomes of the trial

#### INSPIRE intervention: health coaches

This innovative intervention element will include a one-hour orientation/introduction to the program followed by 12 weekly telehealth coaching calls to patients guided by PRO scores and module completion data summarized in the coach’s dashboard. One-hour booster sessions will be delivered at months 5 and 11. The exact roles and expectations of the coaches were determined by current best practices of multiculturally validated programs, patient preferences, and provider/clinic/system needs, with all duties and procedures summarized in a detailed coaching manual [74, 109–115]. We anticipate one PhD-level clinician will serve as the lead coach that supervises 4–5 additional coaches using the UCSF coaching model with a digital patient registry and online dashboard to track participant progress [116].

Coaches will utilize a manual to standardize content facilitation for each week’s materials. Coaches take notes, help patients establish goals, follow their progress, and problem solve issues around intervention engagement and adherence. Coaches were trained for over 80 hours using didactics, role plays, and video review with feedback and remediation when necessary. Coaches will direct participants to their PCP regarding medications, referrals to specialty care, and to address urgent medical issues, and will report AE’s to the study PI and coordinator.

#### Waitlist control condition

Participants randomized into the control condition will continue to receive any interventions from their medical team currently deemed the current standard of care for CP at UCSF/SFHN. Control participants will also receive a printed or pdf manual of the INSPIRE mobile app modules in their preferred language plus one telehealth visit to orient them to the manual. After participants complete the 12-month study, they will be invited to download the INSPIRE app and given access to the INSPIRE intervention website, which they can work through on their own. Participants will not work with a pain management coach due to limited funding to support coaches during and after the RCT. A waitlist control condition was chosen in order to promote participant retention and to address concerns about equity raised by the recruitment clinic directors.

## Outcomes and study measures

### Study measures

**Fig. 3:**
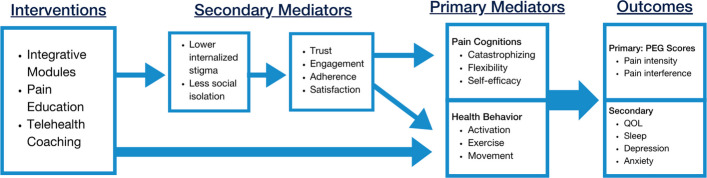
INSPIRE Treatment Effects Pathway Model

Participants will be asked to complete surveys at baseline and at 3, 6, and 12 months. Measures were selected based on the HEAL Common Data Elements and our hypothesized INSPIRE treatment effects pathway model (Fig. 3) [83]. Instruments were translated to Spanish and Chinese (traditional) when necessary and validated when possible. All published measures are cited in Table 1. Adherence with the INSPIRE intervention will be measured with app usage data including module completion rates and log in times, and attendance for coaching sessions. We will collect demographic information including age (in years), race, ethnicity, level of education, employment status, and annual household income as noted in Additional file 4. Other characteristics will include disability insurance, rural-urban commuting area code, and duration of pain.

### Primary outcome

The primary outcome will be Pain, Enjoyment, and General Activity (PEG) score at 3 months. The PEG is a validated 3-item pain assessment tool measuring self-reported pain intensity (P) and interference with enjoyment (E) and general activities (G); items are rated 0–10, with the final PEG score averaging responses over 3 items, with a range of 0–10 [80, 117]. Higher values correspond to worse pain experience and interference.

### Secondary outcomes

Secondary outcomes will include the PEG score at 6 and 12 months, physical functioning, QOL (WHO QOL 2 items; CDE measure), sleep (PROMIS short form sleep disturbance and sleep-related impairment forms), depression (PHQ-8), anxiety (GAD-7), and global satisfaction with treatment (Patient Global Impression of Change) [80–86]. Secondary outcomes will be measured at baseline, 3, 6, and 12 months. Global satisfaction will be measured at 6 and 12 months.

#### Mediators and common factors

Primary mediators (i.e., variables most directly linking the intervention with desired outcomes) will be assessed at timepoints listed in Table 1 and will include pain cognitions (catastrophizing, psychological flexibility, pain self-efficacy) and health behavior (behavioral activation, exercise, movement, sleep) [88–91]. Secondary mediators (i.e., variables that indirectly exert influence on outcomes) include stigma and isolation, trust in healthcare, engagement, and satisfaction with the INSPIRE intervention [92–95]. The hypothesized INSPIRE Treatment Effects Pathway Model is described in Fig. 3.

### Procedures to optimize INSPIRE intervention fidelity and participant adherence

#### Intervention fidelity

##### Mobile App Content

The INSPIRE app was developed and validated by CBT, MBI, MFI, and general pain management experts who were part of the INSPIRE research team [118]. App content was further reviewed and approved by the INSPIRE Clinical Science and Practice Advisory Board to further assess and improve intervention fidelity.

##### Pain Management Coaching

Pain coaches completed 80 hours of training about the INSPIRE intervention including coaching strategies such as motivational interviewing using didactics and mock coaching sessions with experts and peers. Coach competence was measured by expert evaluations of 4 recorded mock coaching sessions (weeks 1, 4, 5, and 6) using a standardized rubric. Those coaches not passing completed remediation by reviewing the sessions, applying the rubric themselves to their and other coaches’ sessions, and continuing practice until attaining a passing score of 80% utilizing the standardized rubric. During the study, 5% of coaching sessions will be randomly selected (via randomization generator) to record and review by intervention experts using a standardized rubric for additional and ongoing fidelity and quality assurance.

#### Participant retention, adherence, and engagement

Given the challenges often faced by our target population and the limitations caused by chronic pain, substantial attention and support will be provided to maximize participant retention and completion of survey instruments at all time points. Text, email, and/or phone call reminders from CRCs will be used to remind participants of assessments and to provide the relevant survey link to REDCap. Non-adherence will be addressed by coaches within the telehealth sessions to better understand and address obstacles to survey completion.

In accordance with Good Clinical Practice (GCP) and International Council for Harmonisation (ICH) guidelines, all participants retain the right to withdraw from the clinical trial at any point. Discontinuation from trial intervention does not mean discontinuation of the study altogether, and the remaining study procedures, follow-up assessment/visits, and data collection will be completed as indicated in the protocol (unless consent is specifically withdrawn).

#### Statistical analysis

We developed a statistical analysis plan for our pragmatic RCT that includes sample size justification, descriptive statistics of our enrolled cohort, primary analysis on our primary outcomes, and supplementary secondary analyses on primary and secondary outcomes.

### Sample size justification

The sample size calculation was based on the primary analysis comparing the primary outcome (PEG scores) between the INSPIRE and control groups at 3 months. Based on the literature, we expect to observe an effect size of 0.3 in PEG score at 3 months [57, 58, 60, 70, 119, 120]. With a power of 0.8 and Type I error of 0.05 in a two-sided independent t-test, assuming 73% retention, a sample size of 586 (293/group) is needed at the enrollment to identify such an effect size at 3 months.

Although we will use Holm’s step-down procedure to adjust for multiple comparisons in secondary analyses, we will take a conservative approach in the power calculation for secondary analyses using Bonferroni with an individual Type I error of 0.01 to control for the family-wise Type I error in 5 comparisons on secondary outcomes. The sample size of 586 (293/group) at the enrollment will provide a power of 0.8 to detect an effect size of 0.366 (a group difference of 0.366*SD) in secondary outcomes between the INSPIRE and control groups. We consider the detectable effect sizes realistic that we expect to observe in the study. With a less conservative Holm’s step-down approach than Bonferroni that we will use in actual secondary analyses to adjust for multiple comparisons, the planned sample size will detect a smaller effect size than 0.366 with a power of 0.80 for secondary outcomes. Adverse events, severe adverse events, and unanticipated problems during the study will be summarized with frequencies and percentages and compared between the treatment groups using Fisher’s exact test or Chi-squared test as appropriate.

### General statistical considerations

Participants’ demographic characteristics (i.e., age, sex, race/ethnicity, socioeconomic status, marriage status) and clinical characteristics at baseline and follow-up will be summarized by intervention groups (INSPIRE vs. control) as well as by sex and language groups to assess potential differences in sex and language groups. Boxplots and other appropriate plots will be used to assess the balance of baseline characteristics between the two groups. The distributions of outcome measures will be checked by histograms and other appropriate plots for the selection of appropriate analysis methods and potential data transformation. Graphs of outcomes over time will be used to determine an appropriate way to model the time effect.

### Primary analysis of the primary endpoint

Our primary analyses will assess the intention-to-treat (ITT) effect of INSPIRE on PEG score compared to the control group at 3 months. The ITT approach will include all the randomized participants, including non-adherers and dropouts, in the analyses. A linear mixed effects model (LMM) will evaluate the group difference over time in change in PEG scores. The models will include the stratification variable (language), group (INSPIRE vs. control), time, and time*group interaction as fixed effects, and random intercept and slope for the nested three-level random effects controlling for correlation due to clustering by clinics, providers, and participants. Baseline demographics and characteristics will be balanced between the two groups by randomization; if any baseline covariates are identified as imbalanced, these will also be included in models. Time will be included in the LMM in an appropriate way (linear, categorical, quadratic, or two pieces) based on the graph of the outcome over time. The time*group interaction will provide the estimated ITT effect of INSPIRE compared to control over time, where the contrast between the INSPIRE and control groups at 3 months is our primary interest, using a significance level of 0.05. The LMM adopts a less stringent assumption on missingness (i.e., missing at random) than complete case approaches (missing completely at random) and will include all available data from all the randomized participants in the analysis.

### Secondary analyses of the primary outcome

Secondary analyses of PEG scores will supplement the primary analysis, including (1) a modified intention-to-treat (mITT) analysis will be performed using the same LMM as the primary analysis but excluding randomized participants who do not participate and drop out after Week 0 and those who do not have chronic pain or have PEG<4 and drop out by Week 1; (2) using an independent *t* test to test corresponding contrasts from the same LMM as the primary analysis to assess if PEG score would be different between the treatment groups at 6 and 12 m; (3) using potential outcome-based approaches to estimate the INSPIRE effects while accounting for nonadherence to intervention [121]; (4) assessing if there are any baseline covariates associated with dropouts and performing sensitivity analyses to see how much the results will change after including covariates associated with dropouts in the models, using multiple imputation, or considering pattern mixture models; and (5) exploring potential heterogeneous effects by demographics or characteristics with sub group analyses. Given the discovery feature of those secondary analyses, a significance level of 0.05 will be used to avoid missing potentially important findings, which will be verified in future studies.

### Analysis of secondary endpoints

Secondary analyses will evaluate the ITT effects of the INSPIRE intervention on physical functioning, QOL, sleep, depression, anxiety, and global satisfaction with treatment with generalized linear mixed effects models (GLMM) like those used for the primary analysis. These GLMM will include language, time, treatment group, time*treatment in fixed effects while controlling correlations by clustering (clinics, providers, and participants). The time*treatment will assess if the secondary outcome changes over time differ by treatment groups. Holm’s step-down procedure will be used to adjust for multiple comparisons in secondary analyses [122].

### Causal mediation analysis

Although the ITT analysis will provide the estimated overall effect of INSPIRE on PEG scores, we will conduct a secondary analysis to understand the pathways of effect for INSPIRE to improve PEG scores around or through intermediate variables using mediation analyses [123–125]. Specifically, a generalized linear model will be fitted for mediators at follow-ups as the outcome and the intervention (INSPIRE vs. control) as the predictor, and then another linear model will be fitted for the outcome PEG reduction over 12 months on the intervention (INSPIRE), mediators, and intervention*mediator interactions with relevant baseline covariates included in the models. Based on the quasi-Bayesian Monte Carlo approximation, estimates from the models will be used to compute the direct and indirect (mediation) effects of the intervention around and through the mediators on CP management [126]. A significant mediation effect will indicate an INSPIRE-mediator-PEG score pathway.

### Data management, entry, coding, security, storage

Data will be collected by system tracking, surveys, and interviews and focus groups related to design, field testing, and post RCT evaluation. Electronic data files will not contain participant identifiers such as names, mailing and email addresses, and telephone numbers. The name and contact information corresponding to each participant will be kept separately in locked cabinets and on secure computers dedicated for project use and will be accessible only to the research staff. All quantitative analyses will be reported as aggregate data. At the study’s conclusion, all computer and paper files with linkage between participant identifiers and database identifiers will be destroyed.

The quality of the data will be monitored regularly on a monthly basis, and on a regular basis by the Learning Health System Oversight Committee. Recruitment and retention statistics will be generated by the PI and CRCs. Data quality will be monitored by random inspection of completed forms and the database by RAs and then the PI. The PI will review the data on a monthly basis to identify any problems with data integrity. The administrative reports will contain no protected health information or allow identification of any participant. The accuracy of data collection procedures and compliance with the study protocol will be maximized through a number of monitoring procedures. The CRCs and data analyst will be trained by Drs. Satterfield, Cheng, and Tsoh who will also be responsible for the development of an operations manual and codebook. The manual will be updated regularly, reflecting field experience with interview, observation, focus group, and intervention protocols and instructions for data reduction and cleaning. Regular staff meetings will keep all study personnel informed of procedural refinements and issues concerning participant contact and protocol implementation. If problems of adherence to intervention protocol are observed, the CRCs will be re-trained.

## Trial oversight and monitoring

Although the risk of serious adverse events is minimal in this health services research study and we will take appropriate measures to ensure confidentiality and appropriate handling of participant data, we are working with potentially vulnerable subjects from stigmatized populations with stigmatized medical conditions. Therefore, we believe it is most prudent to create an internal Data Safety and Monitoring Board (DSMB) comprised of multidisciplinary clinical experts. To address the potential risks of this study, the DSMB will convene at least quarterly to review recruitment as well as monitor the progress of the pragmatic randomized controlled trial and the validity and integrity of study data. Prior to the accrual of participants, a detailed data and safety monitoring plan was submitted and approved by the UCSF Institutional Review Board (IRB).

The DSMB will review all data related to patient risk, adverse events, enrollment, retention, and any other data deemed important to ensure the safety of study participants, the ethical conduct of the study, and the quality of the study. The chair may make specific requests for data and/or confidential interim analyses prior to the DSMB meetings, during the DSMB meetings, or at any other time during the study. The DSMB will also be notified promptly of all adverse events or study events that may threaten patient safety or the integrity of the study. DSMB members may also be called upon for advice in managing such problems. Reports from DSMB meetings will be provided to the NIH program officer. If there are any suspected signs of consistent adverse events, we will ask the UCSF IRB to assist in the appointment of an outside monitor to review data and protocols. Should any other serious and/or recurring unexpected problems arise, the research team will modify the intervention protocol to prevent repetition of such events and, in extreme cases, may recommend termination of the trial. All proposed changes will be evaluated and revised by the DSMB and reviewed by UCSF IRB for approval. The final decision to terminate the trial will be made by the DSMB after consulting with the PI, study team, and the UCSF IRB.

### Reporting of adverse events (AEs), serious adverse events (SAE), and unanticipated problems (UP)

AEs, SAEs, and UPs will be reported by the PI to the UCSF Institutional Review Board (IRB), the DSMB, the NIH Program Officer, and clinicaltrials.gov where the trial will be registered. The UCSF IRB does not play an active role in monitoring data; however, approval from this IRB is necessary to conduct the proposed research, and thus the human subject protections involved in this research will be well-reviewed for safety. Drs. Satterfield, Tsoh, and Aguilera will review adverse events (AEs) and serious adverse events (SAEs) individually in real-time and in aggregate at each DSMB meeting. The PI ensures all protocol deviations, AEs, and SAEs are reported to the IRB and the NIH Program Officer according to the applicable regulatory requirements.

All problems having to do with participant safety will be reported by Dr. Satterfield to the UCSF IRB within three working days. Specifically, the following will be reported in writing: (1) all serious adverse events associated with the study procedures, and (2) any incidents or problems involving the conduct of the study or participant participation, including problems with the recruitment and consent processes. Dr. Satterfield will also provide a full report to the UCSF IRB detailing any problems encountered on an annual basis during years in which the research study is in contact with human participants. In the event the UCSF IRB takes any action pertaining to the study, it will be reported to NIH.

#### Patient, public and shareholder involvement

The INSPIRE Study leveraged Human-Centered Design to ensure active involvement of patients, community members, and stakeholders throughout the development process. Using three iterative rounds of interviews and focus groups, feedback was collected from 8 patients per language group to refine the intervention. The INSPIRE app was developed iteratively with input from these focus groups, interviews, and pilot testing, enabling continuous revisions based on stakeholder feedback. A Community Advisory Board played a key role in providing input, sharing feedback, and reacting to app features, while a separate Science and Clinical Practice Advisory Board, comprised of multidisciplinary pain management specialists, contributed expert guidance. Drafts of proposed interventions were shared during initial meetings, followed by app testing and further refinements based on feedback. The Community Advisory Board also supported recruitment efforts and will be actively involved in dissemination activities.

#### Regulatory, ethics approval, and consent to participate

This study was approved by the Institutional Review Board (IRB) at each participating health care system and registered with ClinicalTrials.gov (NCT06183281). Any necessary protocol modifications will be determined by the PI and co-investigators and submitted for approval by our institutional IRB. All changes will be communicated to all study personnel (and participants if relevant) at team meetings and in written announcements.

#### Dissemination plans

The INSPIRE study is part of the NIH HEAL Initiative—the transdisciplinary effort to stem the opioid health crisis. Key personnel will participate in all appropriate consortium activities, share works in progress, and follow data guidelines described in the HEAL Common Data Elements.

The intervention technology was developed with the goal of dissemination in mind. Our partner, Open Health Network, builds and operates digital services that constitute public infrastructure for the digital era. Moreover, we are following the technology guidelines set forth by Open mHealth to allow sharing with other developers and easy portability of technology innovations.

We will disseminate all research findings through professional conferences and professional publications and welcome collaborations with other institutions. Our data will be made available to other groups interested in similar research or with those doing systematic reviews and/or meta-analyses as well as members of the HEAL consortium. We will use our community partners to disseminate our findings to affected communities and to the lay public.

## Discussion

This protocol describes the rigorous evaluation of the INSPIRE chronic pain intervention. Results may contribute evidence that addresses the viability of using a blended mobile app plus coaching intervention to extend the reach and accessibility of CP interventions while optimizing mobile app engagement. The primary hypothesis states that the INSPIRE intervention will improve PEG scores at 3 months compared to the control group. If this hypothesis is supported, we find that a digital intervention blending CBT, MBT, and MFI with telehealth coaching can efficiently deliver evidence-based, nonpharmacological pain management strategies and effectively improve pain control across diverse populations. If recruitment, retention, and outcomes are consistent across populations, INSPIRE’s process for cultural and linguistic adaptations will have been validated with important implications for improving access to treatment [127]. This intervention could be highly scalable and adaptable to other populations and settings.

Although this pragmatic clinical trial will test the effectiveness of the INSPIRE intervention and the potential mediators of outcomes, we recognize that it will be unable to definitively determine if the intervention effectively improves access to non-pharmacologic pain management, the best strategies for implementation and scaling of the intervention, as well as potential strategies to address clinician bias and stigma. Future implementation work will require a Type 2 or Type 3 hybrid implementation-effectiveness study, and a mixed-methods approach to identify factors to improve scale and acceptability. Additional limitations of this approach include the lack of clinician training to reduce stigma and other potential health care or systemic mediators of pain control. Other studies could consider a permutation of coaching (changing duration, frequency, or using Generative AI), as well as creative ways to promote sustained engagement or even self-study of the intervention.

In conclusion, this NIH-funded 12-month multisite RCT will evaluate the Integrating Nonpharmacologic Strategies for Pain with Inclusion, Respect, and Equity (INSPIRE) intervention that supports patients in combining CBT, MBT, and MFI through a mobile app supported by telehealth coaching. The trilingual intervention has been culturally tailored to meet the needs of HD populations, including Black/African-American patients and other patients speaking English, Spanish, or Cantonese.

## Trial status

This manuscript reflects the INSPIRE protocol version 1.19 approved by the UCSF IRB on 7/21/2025. Participant recruitment began April 28, 2025 and will end at approximately April 20, 2026.

## Supplementary Information

Additional file 1. Trial Registration Data Set.

Additional file 2. SPIRIT Checklist.

Additional file 3. Screenshot of INSPIRE Application Homepage.

Additional file 4. Core Demographics.

## Data Availability

The NIH HEAL Initiative mandates a comprehensive, multi-step data sharing process to ensure that all data generated by HEAL-funded studies adheres to FAIR (Findable, Accessible, Interoperable, and Reusable) principles. This process begins upon receipt of the award and continues through publication, enabling data to be organized, standardized, and accessible for the broader scientific community. All HEAL study-related data will be published on the HEAL data sharing site upon completion of the study and initial analyses.

## Abbreviations

CP: Chronic pain
CBT: Cognitive-behavioral therapy
MBI: Mindfulness-based interventions
MFI: Movement-focused interventions
INSPIRE: Integrating Nonpharmacologic Strategies for Pain with Inclusion, Respect, and Equity
PEG: Pain, Enjoyment, and General Activity tool
SPIRIT: Standard Protocol Items: Recommendations for Interventional Trials
RCT: Randomized Controlled Trial
HD: Health Disparities
HDP: Health disparities populations
B/AA: Black/African American
PT: Physical Therapy
SUD: Substance use disorder
NIH: National Institutes of Health
HEAL: Helping to End Addiction Long-term®
CDE: Common data element
FAIR: Findable, accessible, interoperable, and reusable
F&A: Feasibility and acceptance
PROMIS: Patient Reported Outcomes Measurement Information System
PHQ: Patient Health Questionnaire
GAD: Generalized Anxiety Disorder assessment
TAPS: Tobacco, Alcohol, Prescription medication, and other Substance use tool
UCSF: University of California, San Francisco
SFHN: San Francisco Health Network
SFDPH: San Francisco Department of Public Health
ZSFG: Zuckerberg San Fransico General
CRC: Clinical research coordinator
ICD-10-CM: The International Classification of Diseases, 10th Revision, Clinical Modification
PCP: Primary care physician
PHI: Protected health information
HIPAA: Health Insurance Portability and Accountability Act
BPS: Biopsychosocial
HCD: Human-centered design
PRO: Patient reported outcomes
REDCap: Research Electronic Data Capture
GCP: Good clinical practice
ICH: International Council for Harmonisation
SD: Standard deviation
ITT: Intention-to-treat
mITT: Modified intention-to-treat
LMM: Linear mixed effects model
GLMM: Generalized linear mixed effects model
DSMB: Data Safety and Monitoring Board
IRB: Institutional Review Board
AE: Adverse Event
SAE: Serious Adverse Event
UP: Unanticipated Problem
AI: Artificial Intelligence
CEDAR: Competency Exploration for Development And Readiness
VLMD: Variable-level metadata
DOI: Digital Object Identifier
