# Supplementary material for: Integrating nonpharmacologic strategies for pain with Inclusion, Respect, and Equity (INSPIRE): a digital health study protocol for a pragmatic multisite randomized controlled trial
Source: Trials. 2026 Jan 8;27:109. doi: 10.1186/s13063-025-09402-8 (PMC12882389; doi:10.1186/s13063-025-09402-8)
Supplement: Supplementary file 1 — Additional file 1. Trial Registration Data Set. [file 13063_2025_9402_MOESM1_ESM.docx]

Additional File 1_INSPIRE Trial Registration Data Set

| Data category | Information |
| --- | --- |
| Primary registry and trial identifying number | ClinicalTrials.gov, NCT06183281 |
| Date of registration in primary registry | 8-13-2023 |
| Secondary identifying numbers | [R33NS129050,](https://reporter.nih.gov/quickSearch/R33NS129050) [R61NS129050,](https://reporter.nih.gov/quickSearch/R61NS129050) [R33NS129050](https://reporter.nih.gov/quickSearch/R33NS129050) |
| Source(s) of monetary or material support | National Institutes of Health (R33NS129050) as part of the Helping to End Addiction Longterm (HEAL) initiative |
| Primary sponsor | University of California, San Francisco |
| Secondary sponsor(s) | University of California, Berkeley  National Institute of Neurological Disorders and Stroke (NINDS) |
| Contact for public queries | Tess Fairchild, Tess.Fairchild@ucsf.edu |
| Contact for scientific queries | Jason Satterfield, PhD, Jason.Satterfield@ucsf.edu |
| Public title | Integrating Nonpharmacologic Strategies for Pain with Inclusion, Respect, and Equity (INSPIRE): Tailored Digital Tools, Telehealth Coaching, and Primary Care Coordination |
| Scientific title | Integrating Nonpharmacologic Strategies for Pain with Inclusion, Respect, and Equity (INSPIRE): Tailored Digital Tools, Telehealth Coaching, and Primary Care Coordination |
| Countries of recruitment | USA |
| Health condition(s) or problem(s) studied | Chronic Pain |
| Intervention(s) | Active comparator: Behavioral: CBT, Mindfulness, and Movement delivered through a mobile app and supported by a weekly telehealth pain management coach |
|  | Waitlist Controls: Control participants will receive educational materials about chronic pain and full workbook with non-pharmacologic strategies. Upon conclusion of the study, they are given access to the digital application. |
| Key inclusion and exclusion criteria | Inclusion criteria:  -Age 18 or older  -Speak English, Spanish, or Cantonese  -Have a Primary Care provider at UCSF Health or San Francisco Health Network  -Be willing to use a smartphone (iOS or Android - either their own or one provided by the study)  -Have chronic, non-malignant pain for at least 3 months that causes functional impairment  -Be willing to participate in a 12 month patient-centered chronic pain management study where they will be randomized into one of two comparison arms |
|  | Exclusion criteria:  -Severe mental illness or other condition preventing informed consent  -Chronic pain-related surgery scheduled within the next 12 months |
| Study type | Interventional |
|  | Allocation: randomized intervention model. Parallel assignment masking: single (outcomes assessor) |
|  | Primary purpose: Treatment |
|  | Phase III |
| Date of first enrolment | April 2025 |
| Target sample size | 586 |
| Recruitment status | Recruiting |
| Primary outcome(s) | Change in Pain, Enjoyment, and General Activity (PEG) scores from baseline to 3 months. The PEG is a validated 3-item pain assessment tool measuring self-reported pain intensity (P) and interference with enjoyment (E) and general activities (G); items are rated 0-10, with the final PEG score averaging responses over 3 items, with a range of 0-10. Higher values correspond to worse pain experience and interference. |
| Key secondary outcomes | PEG score at 6 and 12 months, physical functioning/QOL (PROMIS physical function measure, CDE measure), sleep (PROMIS short form sleep disturbance and sleep-related impairment forms), depression (PHQ-8), anxiety (GAD-7), and global satisfaction with treatment (Patient Global Impression of Change). Secondary outcomes will be measured at baseline, 3, 6, and 12 months except Global satisfaction which will only be measured at 6 and 12 months. |
