# Supplementary material for: Integrating nonpharmacologic strategies for pain with Inclusion, Respect, and Equity (INSPIRE): a digital health study protocol for a pragmatic multisite randomized controlled trial
Source: Trials. 2026 Jan 8;27:109. doi: 10.1186/s13063-025-09402-8 (PMC12882389; doi:10.1186/s13063-025-09402-8)
Supplement: Supplementary file 3 — Additional file 3. Screenshot of INSPIRE Application Homepage. [file 13063_2025_9402_MOESM3_ESM.pdf]

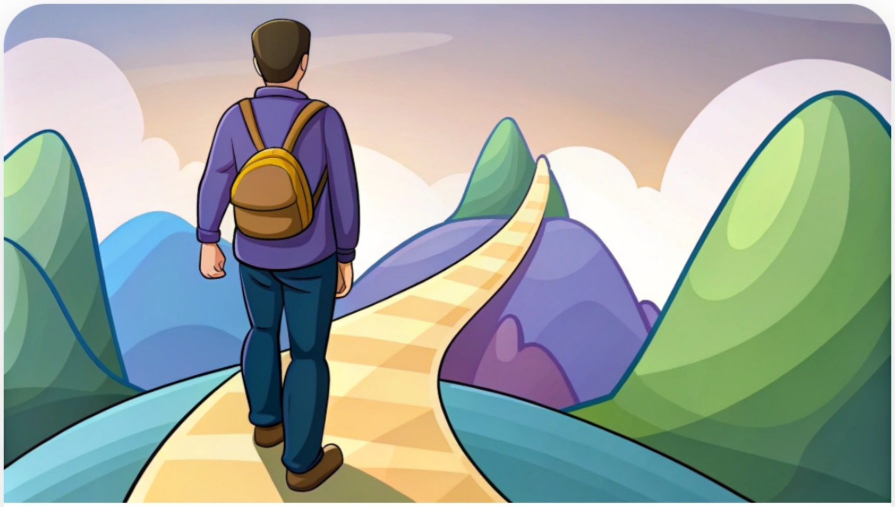

Continue Your INSPIRE Journey

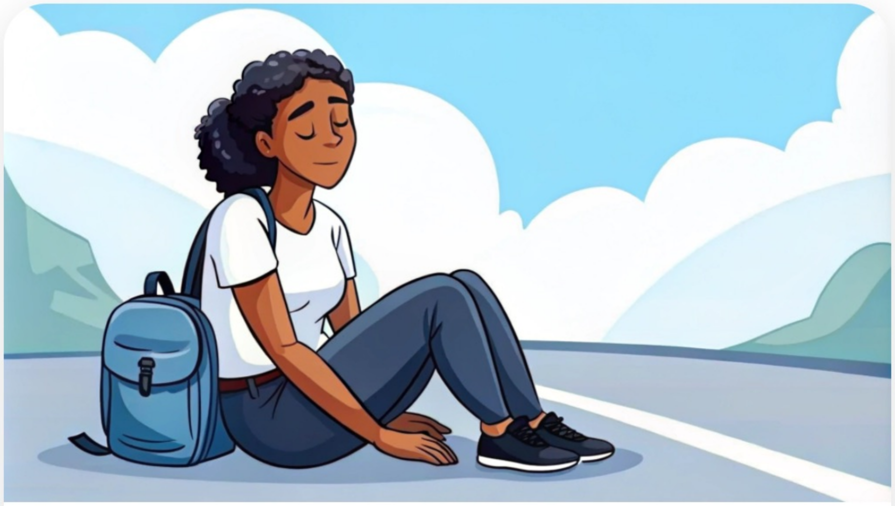

Quick Relief Now

Privacy Policy

Sign Out

Home

My Stuff

Mind

Body

Library
