# Supplementary material for: Integrating nonpharmacologic strategies for pain with Inclusion, Respect, and Equity (INSPIRE): a digital health study protocol for a pragmatic multisite randomized controlled trial
Source: Trials. 2026 Jan 8;27:109. doi: 10.1186/s13063-025-09402-8 (PMC12882389; doi:10.1186/s13063-025-09402-8)
Supplement: Supplementary file 4 — Additional file 4. Core Demographics. [file 13063_2025_9402_MOESM4_ESM.docx]

1. Date of birth: ____________ (mm/dd/yyyy)

2. Age: ____________

3. Sex:

_1. Male _2. Female _3. Unknown _4. Intersex

4. What is your ethnicity?

_Hispanic or Latino

_Not Hispanic or Latino

_Unknown

_Not Reported

5. What is your race? (choose all that apply)

_American Indian or Alaska Native

_Asian

_Black or African American

_Native Hawaiian or Pacific Islander

_White

_Unknown

_Not Reported

6. What is the highest level of education you have completed?

_1. Did not complete Secondary School or Less than High School

_2. Some Secondary School or High School Education

_3. High School or Secondary School Degree Complete

_4. Associate’s or Technical Degree Complete

_6. College or Baccalaureate Degree Complete

_7. Doctoral or Postgraduate Education

7. What is your current employment status?

_1. Full-time employment

_2. Not employed

_3. Part-time Employment

8. What category best describes your current relationship status?

_1. Divorced

_2. Married

_3. Never Married

_4. Separated

_5. Widowed

_6. Domestic Partner

9. What is your annual household income from all sources?

_1. Less than $10,000

_2. $10,000 – $24,999

_3. $25,000 – $34,999

_4. $35,000 – $49,999

_5. $50,000 – $74,999

_6. $75,000 – $99,999

_7. $100,000 – $149,999

_8. $150,000 – $199,999

_9. $200,000 or more

_10. Prefer not to answer

10. Have you ever applied for, or received, disability insurance for your pain condition?

_1. Yes

_0. No

11. How long have you had the type of pain for which you are enrolled in this study?

If one month or more, please list the number of months ________

If less than one month, choose one of the following

_3 weeks (0.75)

_2 weeks (0.5)

_1 week (0.25)

_Less than 1 week (0)

12. In the past year, have you or any family members you live with been unable to get any of the following when it was really needed? Select all that apply.

_a. Childcare

_b. Clothing

_c. Food

_d. Housing

_e. Internet/ Broadband

_f. Phone (e.g., mobile or landline)

_g. Transportation (e.g., private or public)

_h. Utilities (e.g., gas, electric, propane, natural gas, etc.)

_i. Medicine or any health care (medical, dental, mental health, vision)

_j. Other (please specify): _____

_k. Prefer not to answer

*To be completed by study personnel. Refrain from providing participants the following question and information:*

*Only recorded for studies that administer surveys in multiple languages.*

What language was used to administer surveys?

_1. English

_2. Spanish

_3. Swedish

_4. Korean

_5. Japanese

_6. Traditional Chinese

_7. Simplified Chinese

*Study team: Ask for patient’s zip code and report the primary and, optionally, secondary RUCA code.* *RUCA code can be determined based on a HEAL-developed tool, that can be downloaded from the NIH HEAL Common Data Elements Box account using the following link:* *https://nih.box.com/s/mw6p2o3cct2mjp349rnexwua9al3v6v5*

**RUCA code – Primary**: (choose one)

_1. Metropolitan area core: primary flow within an urbanized area (UA)

_2. Metropolitan area high commuting: primary flow 30% or more to a UA

_3. Metropolitan area low commuting: primary flow 10% to 30% to a UA

_4. Micropolitan area core: primary flow within an Urban Cluster of 10,000 to 49,999 (large UC)

_5. Micropolitan high commuting: primary flow 30% or more to a large UC

_6. Micropolitan low commuting: primary flow 10% to 30% to a large UC

_7. Small town core: primary flow within an Urban Cluster of 2,500 to 9,999 (small UC)

_8. Small town high commuting: primary flow 30% or more to a small UC

_9. Small town low commuting: primary flow 10% to 30% to a small UC

_10. Rural areas: primary flow to a tract outside a UA or UC

_99. Not coded: Census tract has zero population and no rural-urban identifier information

**RUCA code – Secondary**: choose one (optional)

1. Metropolitan area core: primary flow within an urbanized area (UA)

_1. No additional code

_1.1 Secondary flow 30% to 50% to a larger UA

2. Metropolitan area high commuting: primary flow 30% or more to a UA

_2. No additional code

_2.1 Secondary flow 30% to 50% to a larger UA

3. Metropolitan area low commuting: primary flow 10% to 30% to a UA

_3. No additional code

4. Micropolitan area core: primary flow within an Urban Cluster of 10,000 to 49,999 (large UC)

_4. No additional code

_4.1 Secondary flow 30% to 50% to a UA

5. Micropolitan high commuting: primary flow 30% or more to a large UC

_5. No additional code

_5.1 Secondary flow 30% to 50% to a UA

6. Micropolitan low commuting: primary flow 10% to 30% to a large UC

_6. No additional code

7. Small town core: primary flow within an Urban Cluster of 2,500 to 9,999 (small UC)

_7. No additional code

_7.1 Secondary flow 30% to 50% to a UA

_7.2 Secondary flow 30% to 50% to a large UC

8. Small town high commuting: primary flow 30% or more to a small UC

_8. No additional code

_8.1 Secondary flow 30% to 50% to a UA

_8.2 Secondary flow 30% to 50% to a large UC

9. Small town low commuting: primary flow 10% to 30% to a small UC

_9. No additional code

10. Rural areas: primary flow to a tract outside a UA or UC

_10. No additional code

_10.1 Secondary flow 30% to 50% to a UA

_10.2 Secondary flow 30% to 50% to a large UC

_10.3 Secondary flow 30% to 50% to a small UC

_99. Not coded: Census tract has zero population and no rural-urban identifier information

Notes:

The rural-urban commuting area (RUCA) codes classify U.S. locations to reflect population density, urbanization, and daily commuting. The classification contains two levels. Whole numbers (1-10) delineate metropolitan, micropolitan, small town, and rural commuting areas based on the size and direction of the primary (largest) commuting flows. These 10 codes can be optionally further subdivided based on secondary commuting flows. Tables to determine RUCA codes from address or zip code are available on the USDA website.

If you want to collect additional/more detailed information on race or ethnicity, you should use the expanded categories from 2024 OMB guidance ([https://spd15revision.gov/content/spd15revision/en/2024-spd15/question-format.html](https://gcc02.safelinks.protection.outlook.com/?url=https%3A%2F%2Fspd15revision.gov%2Fcontent%2Fspd15revision%2Fen%2F2024-spd15%2Fquestion-format.html&data=05%7C02%7Cgiulia.bova%40nih.gov%7C1e59f9c9051e4530b1f408dd1ac1f070%7C14b77578977342d58507251ca2dc2b06%7C0%7C0%7C638696142440412650%7CUnknown%7CTWFpbGZsb3d8eyJFbXB0eU1hcGkiOnRydWUsIlYiOiIwLjAuMDAwMCIsIlAiOiJXaW4zMiIsIkFOIjoiTWFpbCIsIldUIjoyfQ%3D%3D%7C0%7C%7C%7C&sdata=b5iVcKVPBj%2F0lqNgz0yQ4Gepaw8I4ZpT7K85lQ%2F7Dvw%3D&reserved=0)) and ensure that the CDEs can be mapped back to the race and ethnicity CDEs on this demographics form.

Updates in 2024 include: SDOH question added

Updates in 2025 include:

- remove gender identity question from core
- add decimal month options for pain duration less than one month

Reference:

USDA Economic Research Service (2022). Rural-Urban Commuting Area Codes. Economic Research Service, Department of Agriculture. [https://data.nal.usda.gov/dataset/rural-urban-commuting-area-codes. Accessed 2022-05-20](https://data.nal.usda.gov/dataset/rural-urban-commuting-area-codes.%20Accessed%202022-05-20).
